# Assessment of a glycated hemoglobin point-of-care analyzer (A1CNow+) in comparison with an immunoturbidimetric method: a diagnostic accuracy study: Avaliação de um aparelho de diagnóstico local para hemoglobina glicada (A1CNow+) comparado com um método imunoturbidimétrico: estudo de acurácia de teste diagnóstico

**DOI:** 10.1590/1516-3180.2013.9110911

**Published:** 2015-04-14

**Authors:** Aurélie Affret, Luiz Henrique Maciel Griz, Eduarda Ângela Pessoa Cesse, Yuri da Silva Specht, Eduardo Maia Freese de Carvalho, Annick Fontbonne

**Affiliations:** I BSc. Master's Student in Nutrition, Department of Nutrition and Associated Pathologies, Institut de Recherche pour le Développement (IRD), Universités Montpellier, Montpellier, France; II MD, PhD. Professor, Department of Endocrinology, Agamemnon Magalhães Hospital, Universidade de Pernambuco, Pernambuco, Recife, Brazil; III PhD. Epidemiology and Public Health Researcher, Community Health Department, Aggeu Magalhães Research Center, Fundação Oswaldo Cruz (Fiocruz), Pernambuco, Recife, Brazil; IV Undergraduate in Statistics, Community Health Department, Aggeu Magalhães Research Center, Fundação Oswaldo Cruz (Fiocruz), Pernambuco, Recife, Brazil; V MD, PhD. Epidemiology and Public Health Researcher, Community Health Department, Aggeu Magalhães Research Center, Fundação Oswaldo Cruz (Fiocruz), Pernambuco, Recife, Brazil; VI MD, PhD. Epidemiology and Public Health Researcher, Department of Nutrition and Associated Pathologies, Institut de Recherche pour le Développement (IRD), Universités Montpellier, Montpellier, France

**Keywords:** Hemoglobin A, glycosylated, Point-of-care systems, Technology assessment, biomedical, Diabetes mellitus, Primary health care

## Abstract

**CONTEXT AND OBJECTIVE:**

To monitor glycemic control in diabetic patients, regular measurement of glycated hemoglobin (HbA1c) is recommended, but this can be difficult in remote places without access to laboratories. Portable point-of-care testing devices can prove a useful alternative. Our study aimed to assess the performance of one of them: A1CNow+, from Bayer.

**DESIGN AND SETTING:**

Cross-sectional accuracy study conducted at a university hospital in Brazil.

**METHODS:**

We made three successive measurements of capillary HbA1c using the A1CNow+ in 55 diabetic volunteers, while the same measurement was made on venous blood using the hospital reference method (Vitros 5,1 FS). We used the Bland-Altman graphical method to assess the A1CNow+ in relation to the Vitros 5,1 FS method. We also evaluated clinical usefulness by calculating the sensitivity and specificity of A1CNow+ for detecting patients with HbA1c lower than 7%, which is the usual limit for good glycemic control.

**RESULTS:**

The coefficient of variation between repeat testing for the A1CNow+ was 3.6%. The mean difference between A1CNow+ and Vitros 5,1 FS was +0.67% (95% confidence interval, CI: +0.52 to +0.81). The agreement limits of our Bland-Altman graph were -0.45 (95% CI: -0.71 to -0.19) and +1.82 (95% CI: +1.52 to +2.05). The sensitivity and specificity in relation to the 7% limit were respectively 100% and 67.7%.

**CONCLUSIONS:**

Although the A1CNow+ had good sensitivity, its accuracy was insufficient for use as a replacement for laboratory measurements of HbA1c, for glycemic control monitoring in diabetic patients.

## Abstract (pt)

**CONTEXTO E OBJETIVO:**

Para monitorar o controle glicêmico dos diabéticos, é recomendado medir regularmente a hemoglobina glicada (HbA1c). Isso pode ser difícil em locais distantes sem acesso a laboratórios. Uma alternativa é usar aparelhos portáteis à beira do leito do paciente. Nosso estudo visou avaliar o desempenho de um deles: A1CNow+, da Bayer.

**TIPO DE ESTUDO E LOCAL:**

Estudo transversal de acurácia realizado em hospital universitário no Brasil.

**MÉTODOS:**

Medimos, com o A1CNow+, três vezes seguidas, a HbA1c capilar de 55 diabéticos voluntários, enquanto a mesma medida era feita em sangue venoso pelo método de referência do hospital (Vitros 5,1 FS). Usamos a análise gráfica de Bland-Altman para avaliar o A1CNow+ em relação ao Vitros 5,1 FS. Verificamos a utilidade clínica através do cálculo da sensibilidade e da especificidade do A1CNow+ para detectar pacientes com HbA1c abaixo de 7%, limite usual indicando glicemia controlada.

**RESULTADOS:**

O coeficiente de variação entre testes repetidos do A1CNow+ foi de 3,6%. A diferença média entre o A1CNow+ e o Vitros 5,1 FS foi de +0,67% (95% intervalo de confiança, IC: +0,52 para +0,81). Os limites de concordância do gráfico de Bland-Altman foram -0,45 (95% IC: -0,71 para -0,19) and +1,82 (95% IC: +1,52 para +2,05). A sensibilidade e a especificidade em relação ao limite de 7% foram 100% e 67,7%, respectivamente.

**CONCLUSÃO:**

Apesar da boa sensibilidade, o A1CNow+ não tem acurácia suficiente para ser utilizado no monitoramento do controle glicêmico de pacientes diabéticos em substituição das medidas da HbA1c em laboratório.

## INTRODUCTION

Diabetes mellitus is a major public health issue all over the world. In the International Diabetes Federation (IDF) estimates for 2012,^1^ Brazil has a diabetes prevalence of 10.52%, which is higher than in the United States, where the prevalence is 9.35%. The prevalence of self-reported diabetes in the populations of Brazilian state capitals over 18 years of age was 6.3% in 2010.^2 ^From 1950 to 2007, mortality trends due to diabetes increased in most Brazilian state capitals.^3^^,^ 4 Part of this observed increase can be attributed to improvements in access to diagnosis and death certification, but it stresses the importance of improving diabetes prevention and control.^4^

To fight diabetes and other chronic diseases, Brazil is developing strategies within its National Health System (Sistema Único de Saúde, SUS), one of the largest public health systems in the world.^5^ Within SUS, the Family Health Strategy (Estratégia Saúde da Família, ESF) is in charge of most of Brazilian primary care. In 2001, the Brazilian Ministry of Health launched the "Plan for the Reorganization of Care for Arterial Hypertension and Diabetes Mellitus".^6 ^Among the interventions that it supported was regular determination of glycated hemoglobin (HbA1c) levels at ESF primary care units among patients with diabetes. 

HbA1c provides clinicians with an indication of a patient's average blood glucose level over the past two to three months and measurement of this parameter is recommended for assessment of diabetes control. HbA1c also helps estimate the risk of developing diabetes-associated micro and macrovascular complications. This was shown by the results obtained in the Diabetes Control and Complication Trial (DCCT) and United Kingdom Prospective Diabetes Study (UKPDS).^7^^,^ 8

Following these results, the American Diabetes Association (ADA) and other national bodies issuing guidelines for diabetes treatment recommended that one primary goal of therapy should be to maintain HbA1c below 7%.^9^ The ADA also recommended that HbA1c testing should be performed at least biannually in all patients and quarterly for patients whose therapy has changed or who are not meeting treatment goals.^9^

Brazilian recommendations advise one measurement per patient every three months.^6^ However, in many regions of Brazil, these recommendations are difficult to achieve because of a lack of medical analysis laboratories able to meet patients' needs, especially in areas distant from urban centers. This is the case in the state of Pernambuco, in the northeastern region of Brazil, even though the ESF is well established there. Moreover, this situation is not specific to Brazil but can be found in many developing countries where there are remote places with poor access to infrastructure and healthcare professionals. Therefore, HbA1c measurement by means of point-of-care testing (PoCT), directly performed by primary care unit staff, could be a useful alternative to measurement at a medical analysis laboratory. 

## OBJECTIVE 

The aim of this study was to assess the performance of A1CNow+, a PoCT device from Bayer (São Paulo, Brazil), in comparison with the Vitros 5,1 FS method, an immunoturbidimetric laboratory method used in a university hospital in Recife, Pernambuco. Specifically, the objective was to determine whether it could constitute a useful alternative to laboratory measurement, in order to monitor diabetes control in primary care units in remote areas. Our hypothesis was that values of HbA1c given by the PoCT device would be close enough to values given by the immunoturbidimetric method to allow correct decision-making for blood glucose control, in terms of possible modifications of antidiabetic treatments (pharmacological or non-pharmacological). 

## METHODS 

A cross-sectional accuracy study was conducted between June and July 2012 at the endocrinology department of one of the state university hospitals, in Recife, state of Pernambuco, northeastern region of Brazil. Outpatients or inpatients at the department were consecutively approached to be invited to participate, if they were 18 years of age or older, had known diabetes, had a prescription to undergo a venous blood test at the hospital with HbA1c measurement, were not pregnant (for women) and did not have any known hemoglobinopathy, anemia and/or end-stage renal failure. 

Fifty-five patients accepted the invitation to participate. All of them provided informed consent and completed a short questionnaire to gather their baseline characteristics. Then, a venous blood test was performed by a hospital nurse and sent to the hospital laboratory for HbA1c to be measured using the Vitros 5,1 FS method, which was the current HbA1c measurement method used at the hospital and therefore used as the reference against which we evaluated the A1CNow+. The Vitros 5,1 FS method uses venous blood samples and is standardized against the International Federation of Clinical Chemistry (IFCC) reference system for HbA1c and aligned to the DCCT standards through the National Glycohemoglobin Standardization Program (NGSP).^10^

At the same time, their capillary HbA1c was also measured three consecutive times using the A1CNow+, by a master's student who had been trained to perform the method and who had no knowledge of the Vitros 5,1 FS result, since the latter was only returned a few days later. The A1CNow+ is also standardized against the IFCC and aligned to the DCCT standards via the NGSP. It uses both immunoassay and chemistry technology to measure HbA1c and total hemoglobin, respectively, and provides results in five minutes. It is small (length * width * height in mm: 53 * 80 * 15), light and easy to use; it does not require calibration and can be stored at room temperatures up to 50 °C (an important asset for tropical climates, such as in Pernambuco). It is approved for commercialization in Brazil. 

To assess the reproducibility of A1CNow+ measurements, their coefficient of variation (defined as the ratio of the standard deviation to the mean) was calculated using the measurements performed on each patient. For this calculation, only results from patients from whom we obtained at least two valid HbA1c measurements were taken into consideration. We did not use weighted means because of their lack of impact in this study. 

Bland and Altman analysis was used to assess agreement between the two methods.^11^ According to these authors, this analysis requires a sample size of at least 50 units, which determined our choice of number of patients. The differences between the methods (A1CNow+ minus Vitros 5,1 FS) were plotted against the average of the two measurements. The size of the differences and their distribution around zero were tested. 

Finally, to assess the clinical usefulness of A1CNow+, its sensitivity and specificity were calculated in relation to the threshold of 7%, which is the usually recommended limit for diabetes control.^9^ For both the agreement and the usefulness analyses, only the first HbA1c measurement obtained from each patient using the A1CNow+ was used in the calculation, since this is what would happen if the PoCT was used under routine clinical conditions. 

Statistical analyses were performed using Epi Info 3.5.1 and the Statistical Package for the Social Sciences (SPSS), version 19 (SPSS Inc., Chicago, IL, USA). The study was approved by the Ethics Committee of the Aggeu Magalhães Research Center, Oswaldo Cruz Foundation (CPqAM/Fiocruz) and the Brazilian National Commission for Research Ethics (CONEP). All participating subjects were duly informed of the objectives and procedures of the study, and all of them signed an informed consent form. 

## RESULTS 

Most of the patients were women (50 out of 55). Their mean age was 61.3 ± 13.0 years (range: 20-85). Their mean body mass index (BMI) was 27.8 ± 4.2 kg/m^2^, and 75% of the patients were overweight (25 ≤ BMI < 30 kg/m^2)^ or obese (BMI ≥ 30 kg/m^2)^. The mean duration of diabetes was 10.1 ± 8.6 years; 85.5% of the patients were using oral antidiabetic agents and 30.9% were taking insulin injections, alone or combined with oral antidiabetic therapy. 

**Figure 1.**
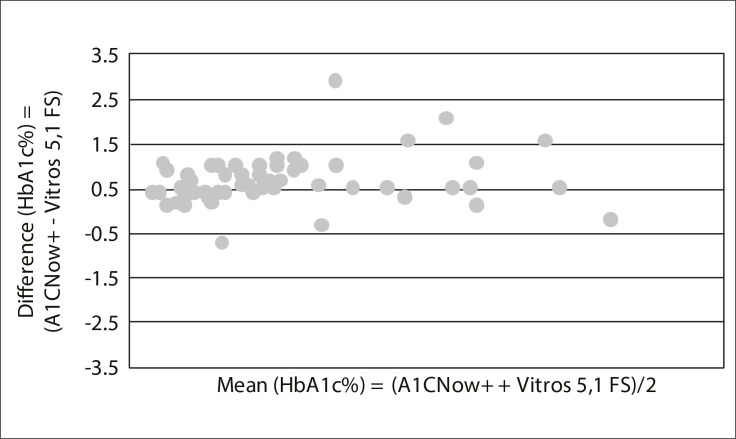
Bland-Altman difference plot comparing the A1CNow+ versus the Vitros 5,1 FS method

From the laboratory method, the mean for HbA1c measurements was 7.26 ± 1.87%. From A1CNow+, the mean for HbA1c measurements was 7.93 ± 1.97%, just considering the first measurement, and 7.91 ± 1.89% considering all measurements. The coefficient of variation for repeated measurements on the same patient was 3.6%. Bland-Altman analysis of accuracy showed that the mean difference between the A1CNow+ and the Vitros 5,1 FS determinations was +0.67% (95% confidence interval, CI: +0.52% to +0.81%) and the standard deviation of the differences was 0.56%, such that A1CNow+ consistently gave higher measurements than the laboratory method (Figure 1). The lower and upper limits of agreement of the graph were -0.45% (95% CI: -0.71% to -0.19%) and +1.79 (95% CI: +1.52 to +2.05). The sensitivity was 100% and specificity was 67.7% in relation to the 7% level (Table 1). It appeared that 18.2% of the patients (10 patients) were "incorrectly classified" using the analyzer. All of them were "false positive": they had HbA1c ≥ 7% with A1CNow+, whereas their HbA1c was < 7% with the Vitros 5,1 FS method. 

**Table 1.** Patients' distribution according to their HbA1c results from the A1CNow+ and Vitros 5,1 FS methods, in relation to the 7% level

		Laboratory method (Vitros 5,1 FS)	
		HbA1c ≥ 7%	HbA1c < 7%
A1CNow+	HbA1c ≥ 7%	24	10
	HbA1c < 7%	0	21

## DISCUSSION 

PoCT has been advocated with the objective, among others, of promoting faster professional decisions and facilitating patients' adherence to medical counseling.^12 ^Indeed, in case of diabetes, studies have shown the benefits of rapid HbA1c results at the time of the patient's visit by improving glucose control through intensification of therapy,^13^^,^ 14 although controversy currently exists regarding this evidence and the analytical quality relative to laboratory testing.^15^

Another potential advantage of PoCT is as a replacement for laboratory measurements in contexts in which laboratory facilities are scarce and often concentrated in urban areas, thus limiting access to populations and jeopardizing quality of care.^16,17^ This is the case in many developing countries, and Brazil, with its continental dimensions, is no exception. 

However, PoCT devices need to be proven to provide sufficiently reliable measurements in order to be recommended for use in cases of lack of or difficulty in access to laboratory services.^18 ^The present study investigated the analytical performance of the PoCT A1CNow+, a device that has been approved for use in Brazil, comparing it with the Vitros 5,1 FS laboratory method. The latter acted as our reference method, since it is the method used in the state university hospital where we conducted this evaluation, which is the reference hospital for diabetes in the state of Pernambuco. 

The coefficient of variation of the A1CNow+ device was found to be 3.6%. This result was in agreement with some previous reports,^19 ^and lower than other results.^20 ^Although it has been recommended that HbA1c assays should have a coefficient of variation of < 2%,^21^ this criterion is very strict and difficult to meet, even for certain laboratory-based methods.^22 ^It would therefore seem inappropriate to impose this goal on PoCT devices measuring HbA1c. Earlier reports recommended that HbA1c assays should have a coefficient of variation of < 5% (ideally < 3%),^23^ in which case our study revealed the A1CNow+ device to be satisfactory in terms of reproducibility. 

However, in terms of accuracy, the Bland-Altman analysis did not demonstrate good agreement between the analyzer and the laboratory method: the results from the A1CNow+ device were systematically higher. The limits of agreement of the graph were -0.45% to +1.79%, thus not fulfilling one of the required NGSP criteria, which is that the 95% CI of the difference between the tested method and the NGSP be ± 0.75% of HbA1c.^10 ^Although one study reported an even greater discrepancy between A1CNow+ results and the reference method,^20 ^others have shown better agreement, with mean differences of between -0.20% and -0.10%, comparing A1CNow+ with the internationally accepted reference laboratory method of high-performance liquid chromatography (HPLC).^19^

In fact, the discrepancy that we found may possibly have been due to the Vitros 5,1 FS method, about which little information is available in the literature. This is indeed the most important limitation of this study: the A1CNow+ results were compared with those obtained from the Vitros 5,1 FS laboratory method and not the internationally accepted HPLC reference laboratory method, which was used in the DCCT and the UKPDS studies.^7^^,^ 8

Nonetheless, it is important to note that we compared A1CNow+ with the laboratory method that was used in the reference hospital for diabetes in Pernambuco, which ensures highly specialized care for diabetic patients from all over the state. Therefore, there is reason to suppose that the hospital would use a high-standard laboratory method for assessing one of the most important parameters for monitoring blood glucose control. 

In keeping with the higher readings in relation to the laboratory method, the A1CNow+ device had good sensitivity (100%). However, its specificity was low (67.7%) in relation to the HbA1c level of 7%. This means that under clinical conditions, use of the A1CNow+ cannot be recommended: the false-positive rate was 10/55 (18.2%), and these patients consequently would have a risk of hypoglycemia if their treatment was intensified using the results from this analyzer. These results were quite similar to those obtained by Arrendale et al.,^24 ^in which the sensitivity was 95% and the specificity was 74%. Accordingly, in addition to the desirability of comparing our results with those of the HPLC method, it might also be interesting in further research to investigate whether A1CNow+ always gives higher results than any laboratory method, in a constant manner. If this were the case, as Arrendale et al. suggested in their study,^24 ^it would still be possible to use this analyzer by applying a correction to the data obtained from it, which would consist of a calculation of the following form: expected laboratory HbA1c = a + b [HbA1c from A1CNow+]. This would be important for diabetic patients living in remote areas with poor access to infrastructure and healthcare professionals, which is often the case in Brazil. These patients would benefit from adequate glycemic control monitoring (HbA1c measurement two to four times a year) when access to venous blood testing in laboratories is difficult. For such purposes, devices like A1CNow+ could become important tools in primary care services.

## CONCLUSION

According to the results from our study, A1CNow+ cannot be recommended for replacing laboratory measurements of HbA1c for glycemic control monitoring among diabetic patients, because the accuracy and specificity of its measurements were insufficient, compared with the method used in a reference university hospital. Further research is needed in order to compare its results with those obtained from a HPLC reference laboratory method, and/or to assess whether it could be used with the aid of a correction equation.
